# Uremic Toxin Indoxyl Sulfate Impairs Hydrogen Sulfide Formation in Renal Tubular Cells

**DOI:** 10.3390/antiox11020361

**Published:** 2022-02-11

**Authors:** Chien-Lin Lu, Chun-Hou Liao, Wen-Bin Wu, Cai-Mei Zheng, Kuo-Cheng Lu, Ming-Chieh Ma

**Affiliations:** 1 School of Medicine, College of Medicine, Fu Jen Catholic University, New Taipei City 242062, Taiwan; 096195@mail.fju.edu.tw (C.-L.L.); 065294@mail.fju.edu.tw (C.-H.L.); wenbin@mail.fju.edu.tw (W.-B.W.); 2 Division of Nephrology, Department of Internal Medicine, Fu Jen Catholic University Hospital, Fu Jen Catholic University, New Taipei City 243089, Taiwan; tch33730@tzuchi.com.tw; 3 Divisions of Urology, Department of Surgery, Cardinal Tien Hospital, New Taipei City 231403, Taiwan; 4 Division of Nephrology, Department of Internal Medicine, Taipei Medical University Shuang Ho Hospital, New Taipei City 235041, Taiwan; 11044@s.tmu.edu.tw; 5 Division of Nephrology, Department of Internal Medicine, School of Medicine, College of Medicine, Taipei Medical University, Taipei 110301, Taiwan; 6 Research Center of Urology and Kidney, Taipei Medical University, Taipei 110301, Taiwan; 7 Division of Nephrology, Department of Medicine, Taipei Tzu Chi Hospital, Buddhist Tzu Chi Medical Foundation, New Taipei City 231405, Taiwan

**Keywords:** chronic kidney disease, hydrogen sulfide, indoxyl sulfate, specificity protein 1, oxidative stress, tubulotoxicity

## Abstract

Hydrogen sulfide (H_2_S) was the third gasotransmitter to be recognized as a cytoprotectant. A recent study demonstrated that exogenous supplementation of H_2_S ameliorates functional insufficiency in chronic kidney disease (CKD). However, how the H_2_S system is impaired by CKD has not been elucidated. The uremic toxin indoxyl sulfate (IS) is known to accumulate in CKD patients and harm the renal tubular cells. This study therefore treated the proximal tubular cells, LLC-PK_1_, with IS to see how IS affects H_2_S formation. Our results showed that H_2_S release from LLC-PK_1_ cells was markedly attenuated by IS when compared with control cells. The H_2_S donors NaHS and GYY-4137 significantly attenuated IS-induced tubular damage, indicating that IS impairs H_2_S formation. Interestingly, IS downregulated the H_2_S-producing enzymes cystathionine β-synthase (CBS), cystathionine γ-lyase (CSE), and 3-mercaptopyruvate sulfurtransferase (3-MST), and these effects could be reversed by inhibition of the IS receptor, aryl hydrocarbon receptor (AhR). As transcription factor specificity protein 1 (Sp1) regulates the gene expression of H_2_S-producing enzymes, we further showed that IS significantly decreased the DNA binding activity of Sp1 but not its protein expression. Blockade of AhR reversed low Sp1 activity caused by IS. Moreover, exogenous H_2_S supplementation attenuated IS-mediated superoxide formation and depletion of the cellular glutathione content. These results clearly indicate that IS activates AhR, which then attenuates Sp1 function through the regulation of H_2_S-producing enzyme expression. The attenuation of H_2_S formation contributes to the low antioxidant defense of glutathione in uremic toxin-mediated oxidative stress, causing tubular cell damage.

## 1. Introduction

Hydrogen sulfide (H_2_S) is a gaseous intracellular signaling transmitter, like nitric oxide and carbon monoxide. The half-life of H_2_S in plasma is less than 30 min, and elimination occurs through excretion in exhaled breath [1] or through binding and oxidation by hemoglobin in the circulation (sulfhemoglobin) [2]. H_2_S can freely move across cell membranes by simple diffusion and does not require a facilitator because its solubility is fivefold greater in lipophilic solvents than in water [3]. In mammalians, H_2_S is synthesized endogenously from L-cysteine through the catalyzation of three major H_2_S-producing enzymes, cystathionine β-synthase (CBS), cystathionine γ-lyase (CSE), and 3-mercaptopyruvate sulfurtransferase (3-MST), along with cystathionine aminotransferase (CAT). CBS and CSE are both dominant enzymes for renal H_2_S generation that are mainly distributed in the proximal tubules and not in the glomerulus or the distal tubules [4]. Compared with CBS and CSE, 3-MST is broadly distributed in the kidneys, including in the proximal tubules, distal tubules, collecting duct, and renal pelvis [5]. As a result, H_2_S generation is abundant in the renal system and plays an important role in renal physiology. H_2_S activates ATP-sensitive K^+^ channels and vasodilates the preglomerular arterioles rather than the post-glomerular arterioles, increasing renal blood flow and glomerular filtration rate [6]. Moreover, H_2_S inhibits tubular Na^+^ transporter activity through pathways such as the Na^+^/K^+^-ATPase and Na^+^/K^+^/2Cl^−^ cotransporters and consequently enhances urinary salt excretion. H_2_S also upregulates aquaporin-2 expression and trafficking in the inner medulla principal cells, increasing the water permeability of the collection duct and promoting urine concentration [7].

The H_2_S level along with the level of renal H_2_S-producing enzymes were shown to both significantly reduce in plasma and remnant kidney tissue in a rat model of 5/6 nephrectomy-induced chronic kidney disease (CKD) [8]. When given NaHS exogenously, an H_2_S donor can ameliorate gentamicin-induced tubulotoxicity in the renal tubular cells by reducing oxidative stress production and apoptosis. Moreover, treatment with H_2_S in CKD rats improves renal function remarkably and alleviates pathological injuries such as tubular dilation and atrophy, interstitial inflammation, and fibrosis [9]. Interestingly, in end-stage renal disease (ESRD), dialysis patients have reduced serum CSE level and H_2_S production, accompanied by high levels of plasma homocysteine and cysteine, which are the substrates for CSE-induced H_2_S biosynthesis. This decrease in H_2_S and subsequent increase in homocysteine are implicated in the pathogenesis of high hypertension prevalence and cardiovascular mortality in dialysis patients [10].

Indoxyl sulfate (IS) is a uremic toxin that accumulates during renal function deterioration. IS exerts its nephrotoxicity effects by directly injuring the renal tubules via transient receptor potential vanilloid 1 (TRPV1) hyperfunction [11] and the induction of the overproduction of transforming growth factor-β1, which participates in the formation of interstitial inflammation and renal fibrosis, both of which that eventually contribute to CKD progression. Additionally, IS increases reactive oxygen species (ROS) production through the promotion of NADPH oxidase 4 (NOX4) [12] and also reduces the activity of glutathione and superoxide dismutase, the powerful antioxidants in renal tubular cells [13]. Therefore, IS greatly increases the oxidative burden that harms renal tubular cells and reduces tubular cell viability.

Until now, no studies have addressed how the H_2_S system is impaired by CKD. We postulate that IS would decrease H_2_S synthesis, which might cause tubular oxidative damage in the cell model of CKD. To investigate the roles of H_2_S- and H_2_S-synthesizing enzymes, the present study examined the impact of IS on H_2_S expression and the subsequent redox alterations in renal tubular cells.

## 2. Materials and Methods

### 2.1. Tubular Cell Culture and Drug Treatment

Because the distal tubules lack the H_2_S-producing enzymes CBS and CSE [4], the present study used Lilly Laboratories cell-porcine kidney 1 (LLC-PK_1_) cells as a model of proximal tubule cell origin, as previously reported [14]. Our recent study showed that LLC-PK_1_ cells are vulnerable to IS [11]. Cells were obtained from the Bioresource Collection and Research Center (Hsinchu, Taiwan). These cell lines were originally derived from the American Type Culture Collection line CL-101 for LLC-PK_1_. All culture media and supplements were purchased from Thermo Scientific HyClone (South Logan, UT, USA). LLC-PK_1_ cells were maintained in Medium 199 supplemented with 3% fetal bovine serum, sodium bicarbonate (1.5 g/L), penicillin (10,000 U/mL), and streptomycin (10,000 μg/mL). The cells were cultured in an incubator, 5% CO_2_ at 37 °C, relative humidity of 95%. Subculture of cells was conducted every three days or have reached about 80% confluence. LLC-PK_1_ cells and 1 mL fresh culture medium were added to each well of the 24-well plate for 2 days. In each experiment, 100 μL culture medium was sampled to mix with chemical treatment for an indicated concentration.

Cells were treated with vehicle solution (0.01% DMSO) or IS (10 mM) for 24, 48, or 72 h. To test the effect of exogenous H_2_S supplementation, two H_2_S donors, NaHS (10, 30, and 100 μM) and GYY-4137 (10, 30, and 50 μM), were treated alone or in combination with IS. To inhibit endogenous H_2_S formation, a non-selective CBS and CSE blocker, aminooxyacetic acid (AOAA, 100 μM), was given alone or in combination with IS. A specific aryl hydrocarbon receptor (AhR) antagonist, CH-223191 (10 μM), was applied to the IS-treated cells to test whether the inhibition of AhR affects the tubular H_2_S system. The dosing of each drug was determined by reference to EC50 or IC50. All chemicals were obtained from Sigma-Aldrich (St. Louis, MO, USA).

### 2.2. Cytotoxicity Assay

The cytotoxic effect of cells treated with or without IS were based on the measurement of lactate dehydrogenase (LDH) released from the injured cells using a Cytotoxicity Detecting kit (Roche Applied Science, Mannheim, Germany). The absorbance spectrum of the final reaction mixture was measured at 492 nm by a standard ELISA reader and LDH concentration was calculated against a standard concentration (Sigma-Aldrich), as previously described [11]. The 3-(4,5)-2,5-diphenyltetrazolium bromide (MTT) assay was used to evaluate the cell viability. At first, treatment with IS alone or in combination with other blockers to LLC-PK_1_ cells for 48 h. After removing the culture medium, the cells were washed twice with PBS (pH 7.4). Next, we added 10 μL MTT (5 mg/mL, pH 7.4) in each well for 4  h, and the optical density (OD) was immediately measured after incubating at 37 °C. The percentage of cell viability was calculated as follows: viable cells (%) = [100 × (treated OD/control OD)], as previously described [11].

### 2.3. Measurement of the H_2_S Level

After treatment with IS alone or in combination of AOAA, 500 μL of cell culture medium was sampled to determine the level of H_2_S. H_2_S production was measured using an ion-selective electrode (Lazar Research Laboratories, Los Angeles, CA, USA) on a Fisher Accumet Model 10 pH meter (Fisher Scientific, Pittsburgh, PA, USA) following the manufacturer’s directions, as previously described [15]. Standards were prepared from NaHS solution with a concentration range of 0.1 to 100 μM.

### 2.4. Western Blot Analysis for Protein Expression

Measurement of the total protein content in the samples was determined by a commercial protein assay kit (Bio-Rad, Hercules, CA, USA). The samples were separated by sodium dodecyl sulfate-polyacrylamide gel electrophoresis (SDS-PAGE) and then electrophoretically transferred onto polyvinylidene difluoride membranes, as previously described [11]. The blotted membrane was blocked in fresh PBS containing 5% non-fat milk and subsequently incubated the membrane in a primary antibodies solution against CSE, CBS, 3-MST, or specificity protein 1 (Sp1) (Santa Cruz Biotechnology, Santa Cruz, CA, USA) overnight at 4 °C. Next, the membranes were incubated for one hour at room temperature with horseradish peroxidase-conjugated secondary antibody (Jackson ImmunoResearch, West Grove, PA, USA) after rinsing with PBS to remove the unbound antibodies. The visualization of the secondary antibody was performed using an enhanced chemiluminescence procedure (Thermo Scientific, Rockford, IL, USA).

### 2.5. Measurement of Sp1 Activity

Nuclear extracted proteins were prepared from the tubular cells using a commercial kit (BioVision, Milpitas, CA, USA), as described above, following the manufacturer’s instructions. Nuclear proteins (10 μg) were used to determine Sp1 activity using a high throughput commercial kit (Abcam, Cambridge, UK). Briefly, a specific double stranded DNA sequence containing the Sp1 consensus binding site (5′-GGGGCGGGG-3′) was immobilized onto a 96-well plate. Active Sp1 was labeled in nuclear extract by oligonucleotide specifically binding Sp1. The epitope of Sp1 is recognized by a primary antibody. Hence, Sp1 can only be detected when Sp1 is in active form or bind to its target DNA. Next, secondary antibody conjugated to horseradish peroxidase (HRP) provides the colorimetric readout at O.D. 450 nm.

### 2.6. Real-Time Quantitative Polymerase Chain Reaction (RT-qPCR) for the Quantification of CBS, CSE, and 3-MST mRNA Expression

Commercial RNA extraction kit (RareRNA, Bio-East Technology, Taipei, Taiwan) was used to isolate cellular RNA, as previously described [11]. The DNase I kit (Invitrogen, Carlsbad, CA, USA) was used to prepare DNA-free RNA solution. In the experiment, 5 µg of total RNA as starting material, 5 µg of oligo(dT)15 primer (Life Technologies, Carlsbad, CA, USA), and 200 units of reverse transcriptase (Moloney murine leukemia virus; Promega, Madison, WI, USA) were used to synthesize complementary DNA (cDNA) at 42 °C for 45 min. Real-time quantitative polymerase chain reaction (RT-qPCR) amplification was conducted using a standard TaqMan PCR protocol on ABI StepOnePlus system (Applied Biosystems, Foster City, CA, USA). The primers used for RT-qPCR are listed in Table 1. All samples were tested in duplicate wells. The ∆Ct (threshold cycle) was calculated by subtracting of the average glyceraldehyde-3-phosphate dehydrogenase (GAPDH) Ct values from the target genes Ct values, which reflects the target gene mRNA level. The change of CBS, CSE, and 3-MST gene expression were calculated as 2^−∆Ct^, and the fold change of genes was presented compared to control group.

### 2.7. Superoxide Formation Examined by Chemiluminescence (CL) Analysis

The release of superoxide (O_2_^−^) into the culture medium was determined as previously described [16]. After treatment for 48 h, 100 μL culture medium was harvested from the treated group instantly. The culture medium was stored at 4 °C in the dark (wrapped in aluminum foil) until measurement. The samples were rinsed with 0.1 mL of PBS (pH 7.4) before CL measurement. During the analysis, culture medium was kept in a dark room in order to detect the emission of photons from CL using Chemiluminescence Analyzing System (CLD-110, Tohoku Electronic Industrial Co., Sendai, Japan). Lucigenin solution (0.1 mM) was prepared in PBS 1.0 mL and added into the culture medium. The area under the curve (AUC) was used to assess the total CL amount. In each experiment, a duplicate test was performed, and the results were expressed as CL counts per second. For further verification of the IS-promoted CL enhancement by superoxide, a separate experiment on cells treated for 48 h with IS was conducted with 50 μL of recombinant bovine superoxide dismutase (SOD, 200 mU) 4 min after lucigenin injection. All chemicals in this assay were obtained from Sigma-Aldrich.

### 2.8. Measurement of Cellular GSH and GSSG Levels

Cellular GSH and GSSG contents were analyzed with a commercial kit (Oxis Research, Portland, OR, USA), and the GSH redox ratio was calculated using the following equation, as indicated by the manufacturer’s instructions: redox ratio (%) = (GSH − 2GSSG)/GSSG × 100. The cell pellet was lysed by treatment with the GSH scavenger supplied in the kit. Cell lysate equivalent to 100 µg of total proteins was added to the assay kit. Cellular GSH and GSSG contents were measured with an ELISA reader at 412 nm based on their individual standard curves.

### 2.9. Statistics

Continuous variables were shown as the mean ± standard error of the mean (S.E.M). Unpaired *t* test or one-way ANOVA was used to assess the differences between each group. The data were analyzed with the Prism 3.0 for Windows (GraphPad Software Inc, San Diego, CA, USA). A two-tailed *p* value < 0.05 was regarded as statistically significant.

## 3. Results

### 3.1. H_2_S Donors Attenuate IS-Induced Cell Damage

**Figure 1:**
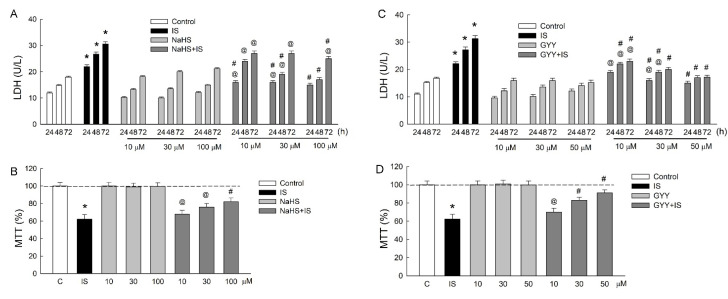
Supplementation with H_2_S attenuates IS-induced tubular damage. The responses to IS and two H_2_S donors NaHS and GYY-4137 (GYY) were evaluated in LLC-PK_1_ cells. (**A**,**C**) The amount of LDH released was determined following treatment with IS or an H_2_S donor, either alone or in combination for 24, 48, and 72 h. (**B**,**D**) Cell viability was examined using the MTT assay after 72 h of treatment. N = 6 for each time-point and treatment. * *p* < 0.05, IS vs. control group; @ *p* < 0.05, NaHS + IS or GYY + IS vs. NaHS or GYY group, respectively; # *p* < 0.05, NaHS+IS or GYY + IS vs. IS group at the same time-point.

We previously showed that IS is toxic for LLC-PK_1_ cells [11]. In this study, we confirmed this and showed that co-treatment with the H_2_S donor NaHS with IS significantly attenuates the release of the cell damage marker LDH (Figure 1A). The dose-dependent effect of NaHS on the reduction in LDH release caused by IS was found to be prominent at the time-points of 24 and 48 h. However, NaHS alone did not affect LDH release. The results of the MTT assay showed that NaHS dose-dependently increased cell viability in the IS-treated cells (Figure 1B).

Using a slow-releasing H_2_S donor, we found that GYY-4137 demonstrated tubuloprotection against IS with a more prominent effect on the reduction in LDH release, especially after 72 h of treatment (Figure 1C). GYY-4137 also showed a similar effect on the improvement of cell viability as that seen with NaHS treatment (Figure 1D).

### 3.2. IS Inhibits Endogenous H_2_S Production

**Figure 2:**
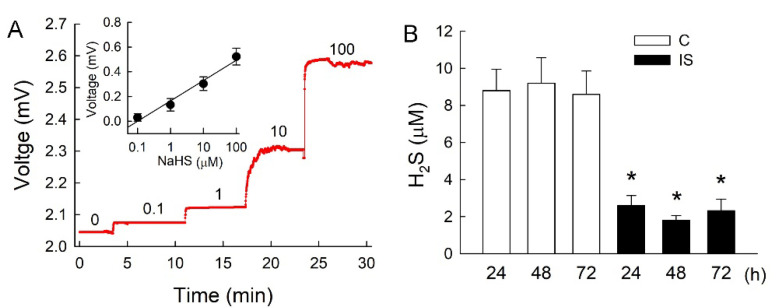
IS impairs H_2_S release in LLC-PK_1_ cells. (**A**) A representative tracing showed changes in the recording voltage (mV) in response to a standard solution of NaHS ranging from 0.1 to 100 μM. The insect graph shows the standard curve of NaHS for six independent determinations. (**B**) Changes in H_2_S levels in culture medium were measured after 24, 48, and 72 h of exposure to the vehicle solution (control, C) or IS treatment. N = 6 in each group and time-point. * *p* < 0.05, IS vs. control (C) group at the same time-point.

Since exogenous supplementation with H_2_S protects tubular cells against IS, the indicated H_2_S formation was impaired in the IS-treated cells. We therefore used an ion-selective electrode to monitor H_2_S release from cells to confirm the tubule damaging effect of IS. The H_2_S donor NaHS was given in a concentration range of 0.1 to 100 μM and demonstrated a stable increase in recording voltage for ~5 min (Figure 2A). The recording voltages correlated well with various concentrations of NaHS (insect in Figure 2A). Interestingly, IS significantly decreased H_2_S release after 24–72 h of treatment when compared with the corresponding controls at the same time points (Figure 2B).

### 3.3. Inhibition of H_2_S Synthesis Aggravates IS-Induced Cell Damage

**Figure 3:**
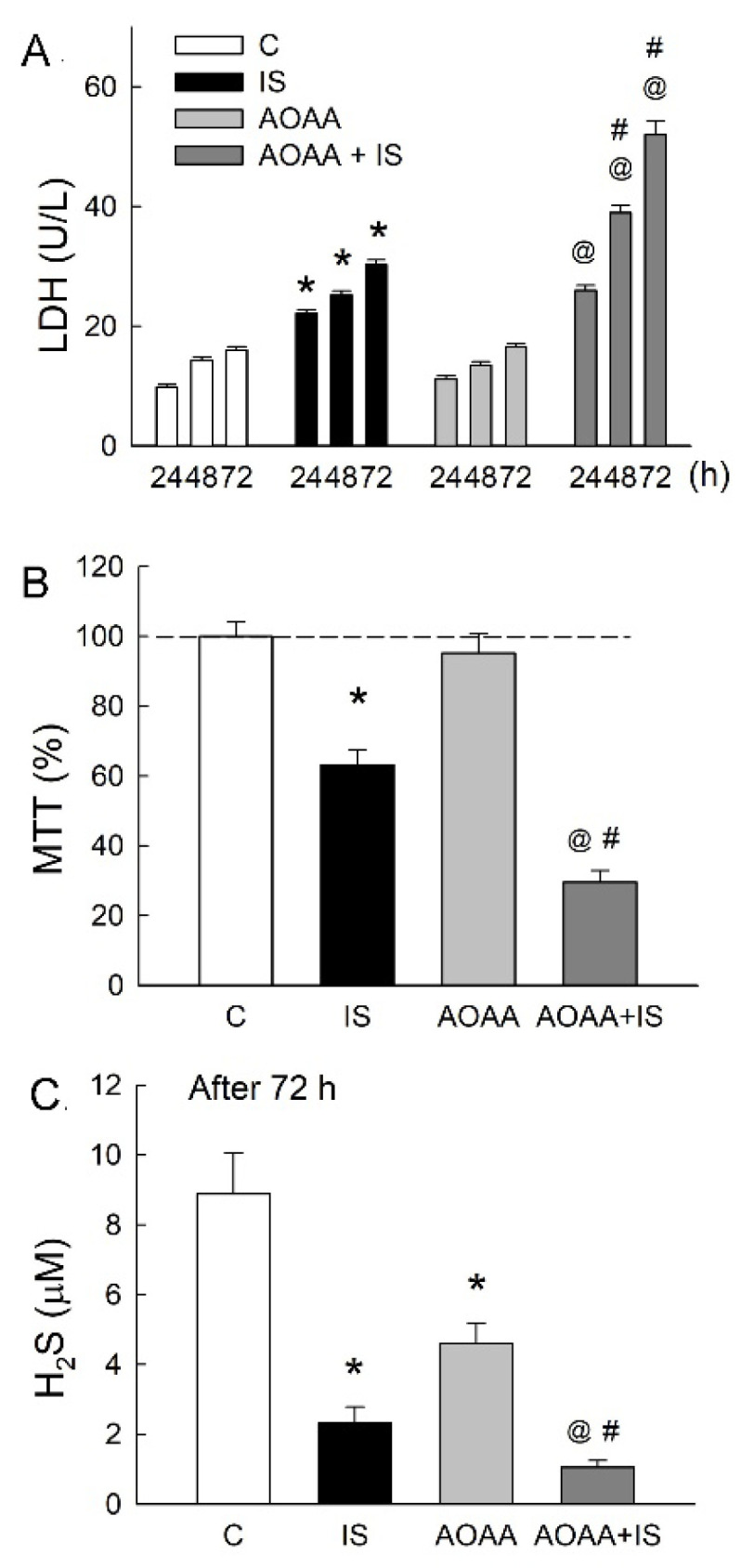
Inhibition of H_2_S-producing enzymes exacerbates IS-induced tubular damage. (**A**) Released LDH was measured under treatment with 10 mM IS or 100 μM AOAA alone or in combination for 24, 48, and 72 h. C, control group treated with vehicle solution. (**B**) The cell viability was examined using the MTT assay after 72 h of treatment. (**C**) H_2_S levels in the culture medium were measured after 72 h of treatment. Note that AOAA markedly attenuated cell survival and H_2_S formation. N = 6 in each group or time-point. * *p* < 0.05, IS vs. control (**C**) group; @ *p* < 0.05, AOAA + IS vs. AOAA group; # *p* < 0.05, AOAA + IS vs. IS group.

We then examined the effects of H_2_S inhibition on cell survival after IS treatment. AOAA, a non-selective H_2_S-producing enzyme blocker, markedly aggravated the IS-induced increase in LDH release and lowering of cell viability (Figure 3A,B). Treatment with AOAA alone showed no effect on cell viability but significantly attenuated H_2_S release when compared with the untreated control group (Figure 3C). Moreover, AOAA decreased H_2_S release more when compared with cells treated with IS only.

### 3.4. IS Attenuates the Expression of H_2_S-Producing Enzymes

**Figure 4:**
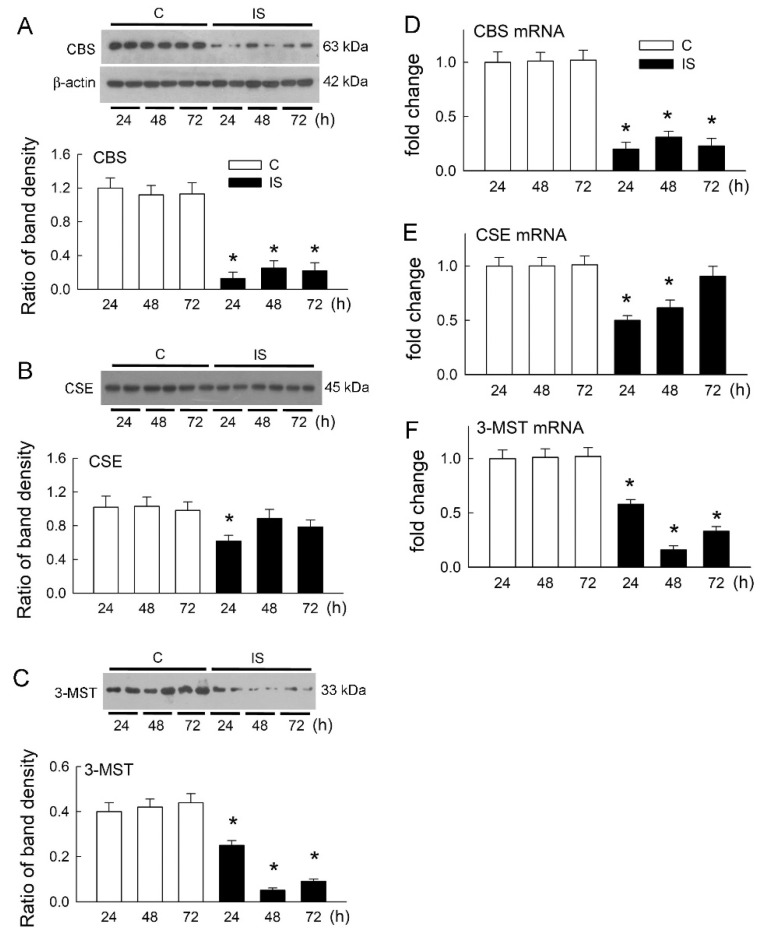
IS lowered the expression of H_2_S-producing enzymes. (**A**–**C**) The representative blots show two independent samples for the protein expression of CBS (**A**), CSE (**B**), and 3-MST (**C**) in cells after 24, 48, and 72 h of treatment with the vehicle solution (C, control) or IS. The lower bar graphs show the ratio of the band density of H_2_S-producing enzymes to β-actin. N = 6 in each group and time-point. (**D**–**F**) The mRNA expression of CBS (**D**), CSE (**E**), and 3-MST (**F**) in control cells and cells treated with IS was examined by RT-qPCR. N = 6 in each group and time-point. * *p* < 0.05, IS vs. control (C) group at the same time-point.

Endogenous H_2_S is produced by CBS, CSE, and 3-MST [17,18,19]. Reduced H_2_S release in the IS-treated cells indicated a defect in H_2_S production. We then tested whether IS affects protein expression in H_2_S-producing enzymes. Interestingly, IS significantly decreased CBS protein levels after 24 to 72 h of treatment when compared with the control group (Figure 4A). However, CSE expression only decreased at the time-point of 24 h after IS treatment and returned to levels similar to those in the controls thereafter (Figure 4B). 3-MST expression was decreased at all time-points after IS treatment and a prominent reduction was seen at 48 h (Figure 4C).

Changes in the mRNA levels of these enzymes were similar to changes in their protein expression. IS significantly decreased CBS mRNA at all time-points (Figure 4D). CSE mRNA was decreased at 24 and 48 h and returned to a similar level as that in the control group after 72 h (Figure 4E). IS significantly reduced the 3-MST mRNA level at all time-points (Figure 4F). Similar to protein expression, a prominent decrease in 3-MST mRNA was found at 48 h.

### 3.5. Blockade of AhR Reverses the Effect of IS on H_2_S-Producing Enzyme Expression

**Figure 5:**
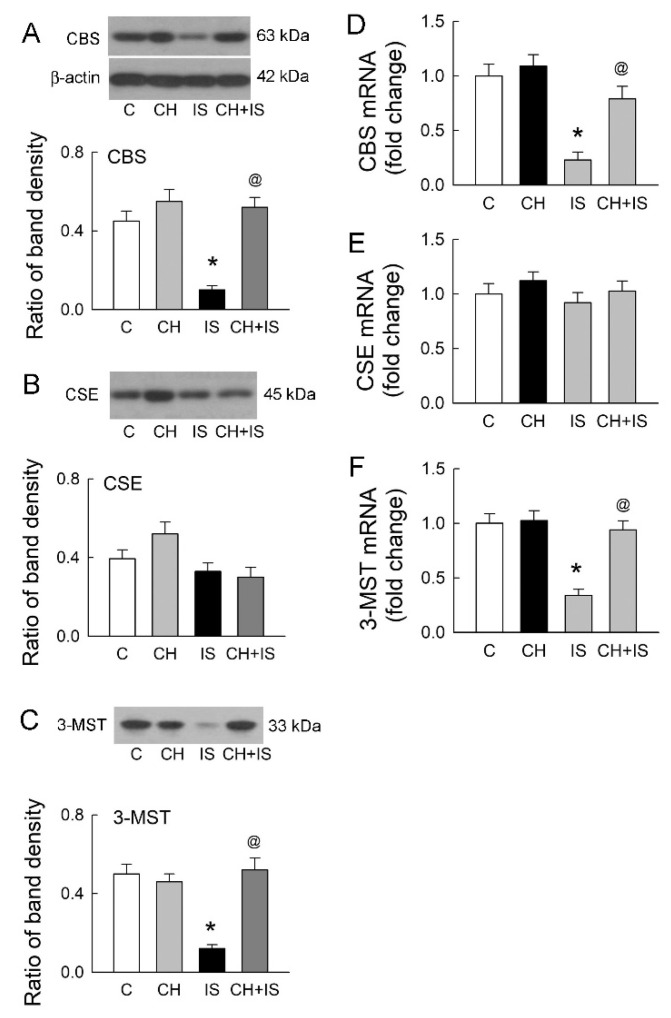
The inhibition of AhR enhances the expression of H_2_S-producing enzymes. (**A**–**C**) The representative blots show one experiment on the protein expression of CBS (**A**), CSE (**B**), and 3-MST (**C**) in cells after 72 h of treatment with vehicle solution (C, control), a specific AhR blocker CH-223-191 (CH), or IS alone or in combination. The lower bar graphs show the ratio of the band density of H_2_S-producing enzymes to β-actin. N = 6 in each group and time-point. (**D**–**F**) The mRNA expression of CBS (**D**), CSE (**E**), and 3-MST (**F**) in cells treated with PBS, CH, or IS alone or in combination was examined by RT-qPCR. N = 6 in each group and time-point. * *p* < 0.05, IS vs. control (C) group; @ *p* < 0.05, CH + IS vs. IS group.

Co-treatment of a specific AhR blocker CH-223191 with IS in tubular cells for 72 h totally abrogated the effect of IS on the reduction in CBS protein expression (Figure 5A). CH-223191 showed no effect on CSE protein expression in cells following 72 h of treatment with IS (Figure 5B). Both CBS and CSE were slightly upregulated in cells following treatment with CH-223191 alone; however, these changes were insignificant. CH-223191 also reversed the effect of IS on the reduction in 3-MST protein expression (Figure 5C).

Changes in the mRNA level after AhR inhibition were similar to changes in protein expression (Figure 5D–F). CH-223191 abrogated the effect of IS on the reduction in both CBS and 3-MST mRNA levels and showed no effect on CSE mRNA.

### 3.6. IS Decreases Sp1 Protein Activity

**Figure 6:**
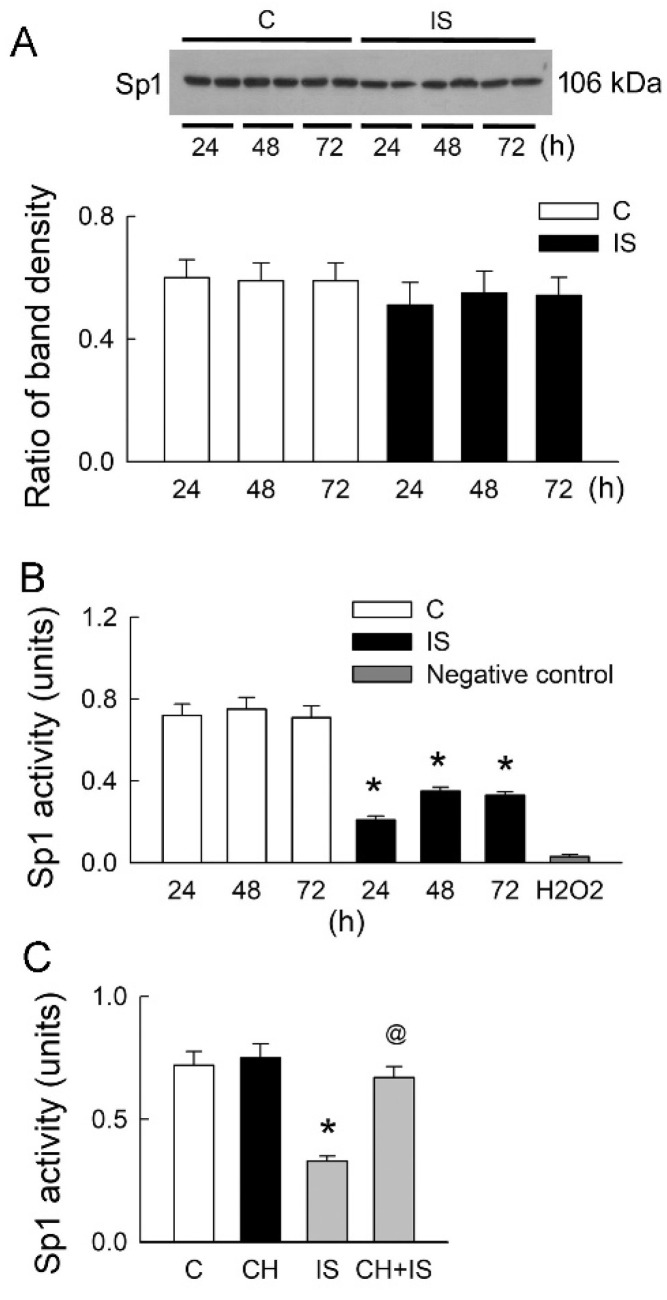
Changes in Sp1 protein expression and activity in LLC-PK_1_ cells. (**A**) The representative blots show two experiments on Sp1 protein expression in cells after 24, 48, and 72 h of treatment with the vehicle solution (C, control) or IS. The lower bar graphs show the ratio of the band density of Sp1 to β-actin. N = 6 in each group and time-point. (**B**) Sp1 protein activity was examined in control cells and cells treated with IS. A control group was treated with 400 μM H_2_O_2_ for 72 h to show the maximal decline in Sp1 activity. N = 6 in each group, treatment, and time-point. (**C**) The protein activity of Sp1 in cells treated with PBS (control), CH, or IS alone or in combination was examined. N = 6 in each group. * *p* < 0.05, IS vs. control (C) group (at the same time-point); @ *p* < 0.05, CH + IS vs. IS group.

The transcription factor Sp1 is known to regulate the expression of H_2_S-producing enzymes such as CBS and CSE [20]. This study further examined whether the effects of AhR blockade on IS-mediated downregulation of H_2_S-producing enzymes are dependent on Sp1. Our results show that IS does not affect Sp1 protein expression after various treatments (Figure 6A). IS, however, significantly attenuated the DNA binding activity of Sp1 (Figure 6B). Interestingly, AhR inhibition totally abolished the effect of IS on the attenuation of Sp1 activity. This clearly indicates that IS-mediated AhR activation affects transcription factor Sp1 binding activity.

### 3.7. Supplementation with H_2_S Increases the Cellular GSH Content

**Figure 7:**
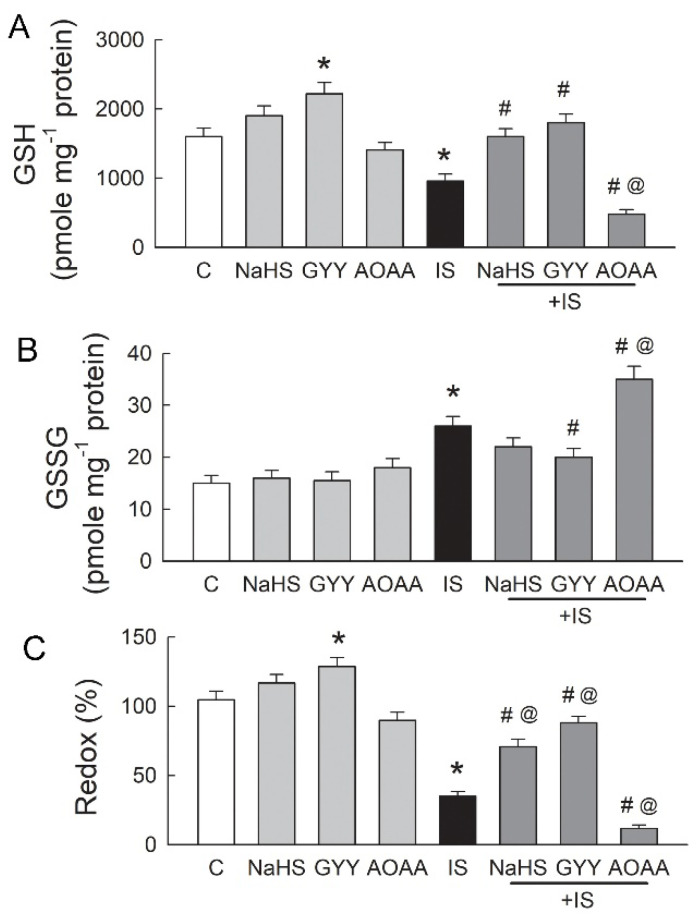
Supplementation with H_2_S reverses the poor redox status caused by IS. (**A**) Cellular glutathione (GSH) content in cells after 48 h of treatment with the vehicle solution (C, control), H_2_S donor NaHS (100 μM), GYY-4137 (GYY, 50 μM), H_2_S inhibitor AOAA (100 μM), or IS in alone or in combination. (**B**) Cellular glutathione disulfide (GSSG) content in cells following the same treatments mentioned above. (**C**) The GSH redox ratio represents the index of antioxidant defense in cells after various treatments. N = 6 in each group. * *p* < 0.05, drug vs. control (C) group; @ *p* < 0.05, drug + IS vs. IS group; # *p* < 0.05, drug + IS vs. drug only group.

H_2_S itself not only functions as an antioxidant by scavenging ROS directly but also enhances the redox ability via glutathione (GSH) upregulation [21,22]. Our results show that the tubular cell content of GSH was lowered after 48 h of treatment with IS (Figure 7A). This effect could be reversed by co-treatment with the H_2_S donor NaHS or GYY-4137 (GYY). Interestingly, the GSH cellular content increased after cells were treated with the H_2_S donor only, and a significant increase in GYY-4137 was found. The inhibition of H_2_S-producing enzymes by AOAA, however, markedly attenuated the GSH content in IS-treated cells. IS also increased the cellular content of glutathione disulfide (GSSG) (Figure 7B). The H_2_S donors NaHS and GYY-4137 lowered GSSG levels in the IS-treated cells, and a significant decrease was found after co-treatment with GYY-4137. A blockade of remnant H_2_S-producing enzyme activity by AOAA significantly increased the GSSG level in the IS-treated cells. We then calculated the GSH redox ratio to estimate the antioxidant status of cells, because this ratio is used as a marker of oxidative stress [23]. Exogenous supplementation with H_2_S by NaHS or GYY-4137 increased this ratio. The highest ratio of 127% was observed in GYY-4137-treated cells, while the ratio was 105% in control cells (Figure 7C). IS significantly decreased the GSH redox ratio to less than half that of controls. Co-treatment of the H_2_S donor NaHS or GYY-4137 with IS significantly increased this ratio. Inhibition of H_2_S-producing enzymes by AOAA in the IS-treated cells largely attenuated the GSH redox ratio to 12%.

### 3.8. Supplementation with H_2_S Donors Attenuates Superoxide Formation Caused by IS

**Figure 8:**
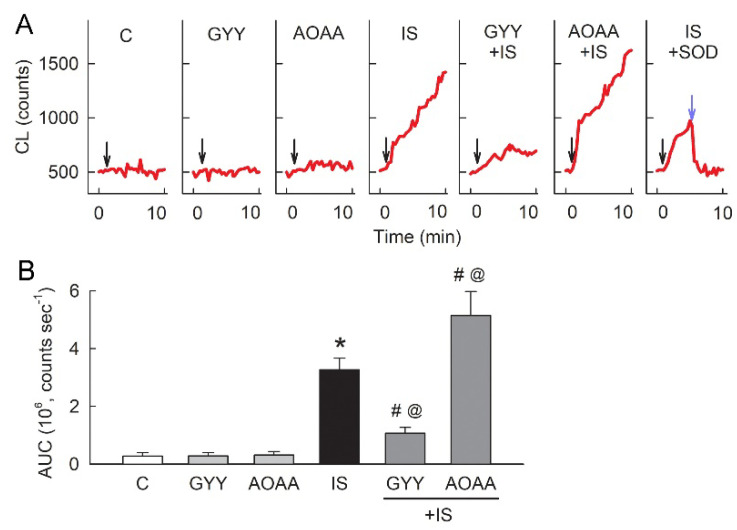
Supplementation with H_2_S reduces IS-induced superoxide formation. (**A**) The representative tracings show changes in chemiluminescence (CL) in the culture medium after 48 h of treatment with the vehicle solution (control, C), 10 mM IS, 50 μM GYY-4173 (GYY), or 100 μM AOAA alone, or in combination. The black arrows indicate lucigenin injection after 1 min of baseline recording. In the rightmost tracing, SOD treatment (200 mU as indicated by a light blue arrow) decreased CL in cells treated with IS for 48 h, which confirms that the CL recordings of lucigenin are derived from superoxide. (**B**) The bar graph shows the total amount of CL as the area under the curve (AUC). N = 6 in each group. Note that IS-induced superoxide formation was attenuated by the H_2_S donor GYY-4173 and enhanced by the inhibition of endogenous H_2_S production via AOAA. * *p* < 0.05, IS vs. control (**C**) group; @ *p* < 0.05, drug + IS vs. IS group; # *p* < 0.05, drug + IS vs. drug only group.

As H_2_S increased the GSH content, we examined whether this effect is associated with oxygen radical formation. Before administration of the probe for detecting superoxide release, lucigenin displayed a basal CL level of around 500 counts (the typical recording tracings in Figure 8A). In cells treated with IS for 48 h, an abrupt increase in lucigenin-enhanced CL, representing superoxide formation, was observed in the culture medium, and this increased gradually during recording. Co-treatment of the H_2_S donor GYY-4137 (GYY) with IS significantly lowered lucigenin-enhanced CL recording. The inhibition of H_2_S-producing enzymes by AOAA markedly enhanced lucigenin-induced CL in IS-treated cells. Recombinant SOD specifically suppressed the CL count after 4 min of lucigenin injection; this confirmed that the increased CL count was associated with superoxide production caused by IS. The results for the area under the curve (AUC) showed that IS increases lucigenin-dependent CL after 72 h of treatment when compared with control cells. Treatment with GYY-4137 or AOAA alone showed no effect on the CL count; however, the CL count significantly decreased and increased, respectively, after co-treatment with IS (Figure 8B).

## 4. Discussion

**Figure 9:**
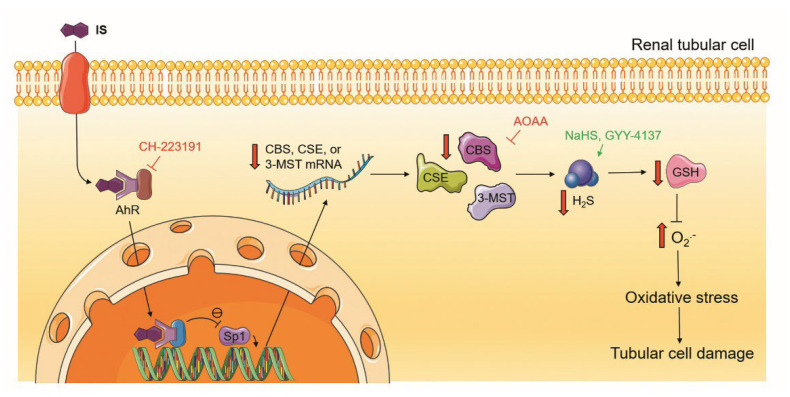
Schematic diagram showing how IS induced renal tubular cell damage. IS was taken up by tubular cells via OATs, as previously reported [11]. IS then decreased the protein activity of transcription factor Sp1. This effect was abolished after the inhibition of AhR via a specific blocker CH-223191, indicating that AhR decreases the DNA binding activity of Sp1. The lowered Sp1 activity probably impaired the protein expression of the H_2_S-producing enzymes CBS, CSE, and 3-MST as well as H_2_S formation. Exogenous H_2_S supplementation through the H_2_S donors NaHS or GYY-4137 ameliorated IS-induced tubular damage by increasing the cellular GSH level and lowering superoxide (O_2_^•−^) release. Inhibition of H_2_S-producing enzymes by AOAA exacerbated tubular injury caused by IS via the enhancement of oxidative stress. Parts of the figure were drawn using pictures from Servier Medical Art. Servier Medical Art by Servier is licensed under a Creative Commons Attribution 3.0 Unported License (https://creativecommons.org/licenses/by/3.0/ (accessed on 1 August 2021)).

As illustrated in Figure 9, IS directly damages renal tubular cells through an increase in oxidative stress. In the proximal renal tubular cells, IS significantly reduces endogenous H_2_S production. Exogenously given H_2_S donors ameliorate IS-induced tubular damage, whereas the impediment of H_2_S generation through the inhibition of H_2_S-producing enzymes exacerbates this damage. These results indicate that defective H_2_S production is implicated in the pathogenesis of IS-induced tubular injury. To elucidate how H_2_S is impaired by IS, we found that IS downregulates the H_2_S-producing enzymes CBS, CSE, and 3-MST, which is part of the mechanism for decreasing H_2_S production in proximal tubular cells. Interestingly, IS also impairs the DNA binding activity of the upstream transcription factor Sp1, which is known to be responsible for the regulation of H_2_S-producing enzyme expression. The deleterious effect of IS on H_2_S-producing enzymes and upstream Sp1 activity can be abrogated by the AhR blocker, which supports the role of AhR in IS-mediated defective H_2_S production and related tubular damage. Furthermore, a lack of H_2_S in IS-treated tubular cells depletes the GSH cell content, as well as increasing superoxide formation, leading to oxidative stress. Exogenous supplementation with H_2_S and endogenous blockade of H_2_S formation to IS-treated cells alleviated and exacerbated oxidative stress, respectively, by superoxide formation.

IS, a protein-bound uremic toxin that is normally eliminated by healthy kidneys, accumulates during renal function deterioration. We previously reported that organic anion transporters (OATs) help the transcellular transport of IS across the cell membranes of the proximal renal tubules [24]. Although this study did not test the effect of OATs on IS uptake, previous studies have provided in vivo evidence that IS and other uremic toxins accumulate in the plasma of OAT knockout mice [25]. Moreover, IS exerts its nephrotoxicity by directly promoting tubular cell death after cell uptake, as we previously reported [26]. Consistent with this, our results show that IS has a direct tubulotoxic effect on LLC-PK_1_. This, however, can be attenuated through the inhibition of AhR. A previous study demonstrated that IS is a strong agonist to human AhR [27]. In the absence of IS, AhR resides in the cytosol as a complex with a dimer of the chaperone heat shock protein 90 (HSP90) [28]. Upon binding to cytoplasmic AhR, the complex of IS/AhR subsequently translocates into the nucleus and dissociates from HSP90 to form a heterodimer with aryl hydrocarbon receptor nuclear translocator (ARNT) as a transcriptional activator. The IS/AhR/ARNT complex binds with the xenobiotic-response element (XRE) sequence in the promoter region of ROS-producing enzyme genes, such as cytochrome P450 enzymes (CYPs), which renders the tubular cells vulnerable to oxidative stress damage. Hence, IS has direct oxidative cytotoxic effects on the renal tubular cells and contributes to the development of tubulointerstitial injury, leading to renal fibrosis, a final stage of CKD [26]. This study consistently showed IS-induced oxidative stress in tubular cells. However, the oxidative stress seen in IS-treated tubular cells was dependent on a weakening of the effect of antioxidant H_2_S.

In hepatic cells, AhR and Sp1 coordinately bind to the XRE and GC box sequence in the proximal promoter region to determine the constitutive expression of CYP1A1, which is responsible for the phase I metabolism of endogenous xenobiotics compounds, such as IS. Notably, AhR bound to the XRE/GC box site can further recruit Sp1 to promote CYP1A1 expression [29]. Our results also demonstrated that there is a protein interaction between AhR and Sp1. Unlike the synergistic effect of AhR and Sp1 in the regulation of CYP1A1 expression, here, we showed that AhR negatively regulates Sp1 activity, as the inhibition of AhR increases Sp1 activity (Figure 6). Sp1 is a zinc finger transcription factor that binds to GC box regions in thousands of genes involved in many cellular processes, including cell differentiation, proliferation, and cell growth [30]. A previous study reported that post-translational modifications of Sp1 activity, including phosphorylation, glycosylation, acetylation, sumoylation, and ubiquitylation, influence its DNA binding affinity and thereby regulate its transcriptional activity, modulating target gene expression [31]. In rat kidneys suffering from ischemia/reperfusion insult, extracellular-signal-regulated kinase 1/2 (ERK)-mediated phosphorylation of Sp1 was shown to be responsible for decreased transcriptional activity of Sp1, leading to a reduction in CBS gene expression [20]. Interestingly, a study reported that AhR also functions in pathways outside its role in detoxification by cross-talk with multiple signal transduction pathways, including those involved in the activation of ERK [32]. Moreover, AhR-mediated oxidative stress also affects Sp1 activity. A previous study showed that hydrogen peroxide (H_2_O_2_) treatment increased the methylation of Sp1 and repressed Sp1 transcriptional activity [33]. Further study is required to identify the roles of ERK and H_2_O_2_ on Sp1 function in terms of the regulation of H_2_S-producing enzyme expression after IS treatment.

In human embryonic kidney cells, the CSE gene promoter displayed a high binding affinity for Sp1 [34]. In human smooth muscle cells of the aorta, there are Sp1 consensus binding sites present in the core promoter of the human CSE gene, and H_2_S production by CSE is essential for the maintenance of the smooth muscle cell phenotype [35]. Moreover, transactivating roles for Sp1 and nuclear factor Y play an essential role in regulating CBS activity. The alteration of Sp1 phosphorylation or Sp1 synergism with nuclear factor Y influences the differential binding of Sp1 to the CBS promoter and then affects CBS gene expression [36]. Therefore, Sp1 is a regulatory factor that modulates H_2_S-producing enzyme expression and H_2_S production to make a cell-specific pattern of H_2_S renoprotection. In addition to the transcriptional effect of Sp1, CSE activity for H_2_S production has been reported to be affected by changes in intracellular Ca^2+^ ([Ca^2+^]i) directly, where an increase in [Ca^2+^]i enhances CSE activity and vice versa [37]. We previously showed that IS-mediated AhR activation can induce a possible [Ca^2+^]i overload via hyperfunction of the Ca^2+^-permeable TRPV1 in tubular cells [38]. [Ca^2+^]i overload-mediated by TRPV1 to reduce CSE activity needs further study. Similar to CSE, an increase in [Ca^2+^]i also attenuates the activity of the 3-MST/CAT pathway for H_2_S production, and the enzyme regulated by Ca^2+^ has been suggested to be CAT and not 3-MST [39]. Furthermore, oxidative stress significantly suppresses 3-MST activity via the enhanced oxidation of three redox-sensitive cysteines (Cys154, Cys247, Cys263) in the catalytic site of 3-MST, reducing H_2_S production [40]. As the present results show that oxidative stress is associated with IS/AhR-mediated tubular damage by impeding H_2_S formation, a detailed study is required to see whether this is dependent on the increased oxidation of 3-MST.

Alteration of H_2_S has been implicated in various kidney diseases, including renal ischemia/reperfusion injury (IRI) [41], hypertensive kidney injury [42,43], diabetic nephropathy [4,44], obstructive kidney injury [45], and CKD progression [6,8]. IRI is the most well-studied model to demonstrate the impact of altered H_2_S production on the renal tubules. In the IRI model of the rat kidneys, both CSE and CBS protein levels are decreased at 4 days after ischemia, and this persists throughout this period for up to 21 days. Impaired H_2_S production significantly increases formation of the phosphorylated form of histone H2A (pγH2AX), which indicates that oxidative damage to DNA occurs in renal tubular cells in response to IRI. Instead, exogenous supplementation CSE-deficient mice with H_2_S attenuates the area of renal cortical necrosis, reduces the quantity of pγH2AX-positive cells, retards the renal function decline, and rescues animal survival [46]. In this study, we consistently showed that the deleterious effect of IS on tubular cell damage can be significantly reversed by the H_2_S donors NaHS or GYY-4137. H_2_S deficiency, therefore, increases the vulnerability of the renal tubules to oxidative damage.

Previous studies showed that the mechanisms underlying IS-induced tubular oxidative damage include increases in NOX4 activity and ROS generation [47], downregulation of Nrf2 through NF-κB activation, a subsequent reduction in the expression of the antioxidant HO-1 [48], and decreases in the activity of GSH and SOD, which are responsible for defense against oxidative stress in the renal tubules [49,50]. However, there is no available literature that discusses the role of H_2_S in IS-mediated tubular damage. In fact, H_2_S and its dissociated ion HS^-^ act as a powerful one-electron chemical reluctant and have a high capacity to scavenge ROS by directly reacting to superoxide and H_2_O_2_ [51,52]. In this study, we consistently showed that exogenous supplementation with H_2_S attenuates superoxide formation in the IS-treated tubular cells. In addition to H_2_S itself, a previous study demonstrated that H_2_S increases the intracellular GSH level by enhancing the production of the cystine/cysteine transporter and redistributes GSH to the mitochondria, where a large amount of ROS is produced [22]. In this study, we also showed that exogenous supplementation with H_2_S increases the intracellular GSH content in IS-treated tubular cells. Hence, H_2_S, together with GSH recruitment, act as an overall antioxidant defense against oxidative damage triggered by IS/AhR signaling. Accordingly, impaired H_2_S production by IS renders tubular cells vulnerable to oxidative stress damage and underlies one of the mechanisms of IS-mediated tubulotoxicity, which inevitably leads to tubulointerstitial inflammation and renal fibrosis.

The single cell line used is the limitation of our study. LLC-PK_1_ is the most widely used model to study the harmful uremic toxins on renal tubular cells [11,49,53,54]. Certainly, additional proximal renal tubular cell lines or in vivo rat CKD model should be considered to provide consistency proofs to explore the deleterious effect of IS on renal tubular damage.

## 5. Conclusions

Our results show that treatment of the proximal tubule cells with IS impairs H_2_S formation and induces tubular oxidative damage. Downregulation of the H_2_S-producing enzymes CBS, CSE, or 3-MST and a decrease in Sp1 DNA binding activity underlie the mechanism behind the decrease in H_2_S production in the proximal renal tubules. As expected, AhR is involved in IS-mediated defective H_2_S production and subsequent renal tubular damage. The accumulation of IS during CKD progression impairs H_2_S formation, rendering tubular cells more susceptible to oxidative injury. Thus, H_2_S may serve as a potential therapeutic molecule to alleviate renal function decline in CKD.

## Figures and Tables

**Table 1:** List of primer sequences used for RT-qPCR.

Gene	GenBank Accession Number	Sequence
CBS	XM_039080137	5′-TAG ACG GCA GAG CCT TTC GA-3′ (forward) 5′-AAT CCC CGG CCG TAG AAC-3′ (reverse)
CSE	NM_017074	5′-ACA CTT CAG GAA TGG GAT GG-3′ (forward) 5′-TGA GCA TGC TGC AGA GTA CC-3′ (reverse)
3-MST	NM_001013440	5′-CTG GGA AAC GGG GAG CG-3′ (forward) 5′-GCT CGG AAA AGT TGC GGG -3′ (reverse)
GAPDH	XM_039097338	5′-TTA GCA CCC CTG GCC AAG G-3′ (forward) 5′-CTT ACT CCT TGG AGG CCA TG-3′ (reverse)

## Data Availability

Data are contained within the article.

## Abbreviations

3-MST	3-mercaptopyruvate sulfurtransferase
AhR	Aryl hydrocarbon receptor
AOAA	Aminooxyacetic acid
ARNT	Aryl hydrocarbon receptor nuclear translocator
CAT	Cystathionine aminotransferase
CBS	Cystathionine β-synthase
CKD	Chronic kidney disease
CSE	Cystathionine γ-lyase
CYPs	Cytochrome P450 enzymes
ERK	Extracellular-signal-regulated kinase
ESRD	End-stage renal disease
GAPDH	Glyceraldehyde-3-phosphate dehydrogenase
GSH	Reduced glutathione
GSSG	Glutathione disulfide
H_2_S	Hydrogen sulfide
HSP90	Heat shock protein 90
IRI	Ischemia/reperfusion injury
IS	Indoxyl sulfate
NF-κB	Nuclear factor-κB
Nrf2	Nuclear factor erythroid 2-related factor 2
OATs	Organic anion transporters
ROS	Reactive oxygen species
SOD	Superoxide dismutase
Sp1	Specificity protein 1
TGF-β1	Transforming growth factor-β1
TRPV1	Transient receptor potential vanilloid 1
XRE	Xenobiotic-response element
